# Strengthening capacity to use research evidence in health sector policy-making: experience from Kenya and Malawi

**DOI:** 10.1186/s12961-019-0511-5

**Published:** 2019-12-19

**Authors:** Rose N. Oronje, Violet I. Murunga, Eliya M. Zulu

**Affiliations:** 1 African Institute for Development Policy (AFIDEP), P.O. Box 14688-00800, Westlands, Nairobi, Kenya; 2 African Institute for Development Policy (AFIDEP), Lilongwe, Malawi

**Keywords:** Evidence-informed policy-making, research use capacity, research use, evidence-informed decision-making, research translation, health sector policy-making, institutional capacity for research use

## Abstract

**Background:**

Among the many barriers to evidence use in decision-making, weak capacity for evidence use has attracted a lot of focus in the last decade. The study aims to inform and enrich ongoing and future efforts to strengthen capacity for evidence use by presenting and discussing the experiences and lessons of a project implemented in Kenya and Malawi to strengthen individual and institutional capacity for evidence use within the ministries of health (MoHs).

**Methods:**

This paper draws on the internal and external evaluations of a 3-year project funded by the United Kingdom’s Department for International Development, the Strengthening Capacity to Use Research Evidence in Health Policy (SECURE Health). To strengthen individual capacity, the project implemented a training and mentorship programme for 60 mid-level policy-makers in the two MoHs. To strengthen institutional capacity, the project conducted sustained advocacy with top leaders to strengthen structures that enable evidence-informed decision-making (EIDM), supported Kenya to develop research-for-health policies and priorities, supported Malawi to review the implementation of its health research agenda, developed EIDM guidelines for both MoHs, and supported bi-annual evidence dialogues to improve interactions and raise the profile of evidence. Internal evaluation included baseline and endline surveys (93 baseline and 92 endline interviews), 60 in-depth interviews, and intervention-specific evaluations (pre–post tests for training workshops, feedback forms for policy dialogues and tracking effects of advocacy efforts). The external evaluation was implemented alongside project implementation, conducting three annual evaluations.

**Results:**

The results show that training and mentorship programmes in EIDM were effective in improving competencies of civil servants. However, such programmes need to train a critical mass to be effective in enhancing EIDM practice at the MoHs. On strengthening institutional capacity for EIDM, while the project achieved some success, it did not realise long-lasting effects because of its limited time of implementation and limited focus on sustained political economy analysis, which meant that the intervention was negatively affected by frequently changing interests within the MoHs.

**Conclusions:**

Although training and mentorship are effective in improving EIDM competencies, they need to be incorporated in existing pre-service and in-service training programmes for sustainability. Strengthening institutional capacity for evidence use is complex and needs sustained political commitment and long-term investments.

## Key messages

- Although strong individual and institutional capacities are critical in enabling evidence-informed decision-making (EIDM), these remain weak in many developing countries for many reasons, including lack of EIDM training programmes for civil servants, and low priority and investments in strengthening institutional structures and mechanisms for enabling EIDM.
- Training and mentorship programmes in EIDM are effective in improving competencies of civil servants, but these need to be incorporated in existing pre-service and in-service training programmes to be sustainable and ensure the training of a critical mass.
- Strengthening institutional capacity for evidence use is complex and needs sustained political commitment and long-term investments.

## Background

Evidence has an important role to play in improving policy, programme and practice decisions that ultimately improve development effectiveness [1–5]. Even then, it is widely acknowledged that policy-making is a complex, political process, with evidence being but one of the factors that shape or inform it [6, 7]. Because of this appreciation, there has been sustained momentum in development efforts to better understand effective ways through which evidence could have a greater bearing on development policy and programme decisions and implementation. Among the many barriers to evidence use, weak capacity for evidence use in policy and programme decisions has attracted a lot of focus in the last decade [4, 8–12]. This paper uses the broad definition of capacity provided by the United Nations Development Programme (UNDP) as “*the ability of ‘individuals, institutions and societies’ to perform functions, solve problems, and set and achieve objectives in a sustainable manner*” [13]. Capacity for research use in this paper has to do with interventions that improve the ability of both individuals and institutions to promote and facilitate research use in policy and programme decisions, i.e. evidence-informed decision-making (EIDM).

Potter and Brough [14] note that capacity development at institutional level involve such processes that are sustained over time and not as easily derailed by changes in individual staff, or structures that ‘institutionalise’ these processes across a wide range of decision-making stakeholders; it also concerns governance systems and incentives. At individual level, capacity concerns technical skills and abilities as well as the attitudes, motivation and ability to assume desired behaviours that promote and apply evidence [15]. Accordingly, at both institutional and individual levels, capacity goes beyond technical issues. All these point to complexity, which means that strengthening capacity for evidence use in already complex policy-making systems [16, 17] needs to take a comprehensive focus on institutions, individuals and society, with a deep understanding of the political economy driving or undermining change within contexts [7, 12, 18].

A number of documented capacity development efforts have been implemented with the aim of increasing or enhancing the use of evidence in decision-making. A systematic review by Clar et al. [19] in 2011 on the effectiveness of interventions aimed at improving the use of health research into policy and practice in low- and middle-income countries, found 17 interventions (out of a total of 25) to have included an element of training or capacity-building as an intervention for enabling evidence use. Even then, of the 17 interventions, only 4 specifically included building the capacity of policy-makers and policy-influencers to understand and use research in their work.

Hawkes et al. [12] conducted an evaluation of five multi-country interventions (implemented in Bangladesh, Gambia, India and Nigeria) aimed at strengthening capacity for evidence use. The interventions focused on all the three levels of capacity-building (i.e. individual, organisational and institutional). At the individual level, their results showed that the interventions were successful in building the capacity of individuals to access, understand and use evidence/data. At the organisational level, their results showed that the interventions implemented, which mainly involved support to infrastructure (e.g. through information technology resources), were also successful. At the institutional level, they found that there was less interest to address the need to strengthen institutional capacity; however, this was acknowledged to be fundamental to promoting sustainable use of evidence and was recognised as requiring resources, legitimacy and regulatory support from policy-makers. Hawkes et al. [12] noted the lack of focus on the political environment as a major weakness in these interventions since policy-making is inherently political and understanding interventions that enable evidence use need to also understand the political environment and its bearing on the interventions.

Vogel and Punton [18], in their evaluation of the six Building Capacity to Use Research Evidence (BCURE) projects funded by the United Kingdom’s Department for International Development (DFID), found that while the projects made notably good progress in building individual capacity for evidence use and providing tools for promoting and enabling evidence use, they did not contribute to lasting institutional capacity development. They argued that this was largely because the projects had a “*superficial good fit*” with government agendas but lacked a deeper understanding of the political economy dynamics within government ministries that shape the change [18]. They concluded that “*working with governments to build capacity for evidence use requires a politically informed and multidimensional approach*” ([18], p. iv).

The literature above points to the need for a better and nuanced understanding of efforts to strengthen institutional capacity for evidence use as well as understanding context-specific lessons and insights in building institutional capacity for evidence use. This is especially important given the increasing focus on strengthening institutions (see [9, 20]) in order to inform future interventions.

This paper discusses our experiences and lessons from one of the BCURE projects implemented in Kenya and Malawi to strengthen capacity for evidence use within the ministries of health (MoHs). The intervention implemented simultaneous activities to build technical skills at individual level, strengthen institutional policies and structures that enable evidence use, and cultivate a supportive political environment for enabling increased demand and use of evidence in health policy-making in Kenya and Malawi. This paper discusses fresh, context-specific lessons and insights in strengthening institutional and individual capacities in government agencies for evidence use in Kenya and Malawi. In this way, the paper enriches the emerging literature on strengthening institutions for evidence use. Although our BCURE intervention focused on both MoHs and parliaments, this paper focuses only on the experiences and lessons from the MoHs in the two countries.

## Methods

### Data collection

This paper draws primarily from the internal and external evaluation of our BCURE intervention, the Strengthening Capacity to Use Research Evidence in Health Policy (SECURE Health) project. The SECURE Health project aimed to optimise individual and institutional capacity to use research in health sector decision-making in Kenya and Malawi.

#### Internal monitoring and evaluation of the intervention

The intervention’s internal monitoring and evaluation comprised two components. The first was the baseline and endline assessments of capacity needs. The baseline and endline assessments were cross-sectional descriptive studies that used both quantitative and qualitative methods. Data were collected through semi-structured questionnaires completed in face-to-face interviews and in-depth interviews. With regards to sampling, the MoH Permanent/Principal Secretary and all heads of directorates (i.e. top-level policy-makers) in both MoHs were interviewed (Kenya MoH had 5 directorates whereas Malawi MoH had 7 directorates). In addition, we sampled at least 2–3 technical staff (i.e. mid-level policy-makers) from all divisions and units under the directorates (national-level MoH). This was done to ensure representativeness of the data gathered. The selection of the 2–3 staff for interview was informed by this criterion – all division/unit heads were to be interviewed and they would then advise on two relevant staff from the division/unit who should also be interviewed. In cases where the division/unit heads were unavailable, we engaged the staff at the MoH research division/department to identify the relevant staff in the division/unit to be interviewed. We also purposively sampled and interviewed senior health researchers in either leadership positions in research institutions or those actively involved in the work of MoH policy task-forces or technical working groups. In-depth interviews with the researchers focused on understanding their experiences with supporting health research use and their perceptions of the status of institutional capacities for enabling EIDM.

Table 1 below provides the samples interviewed in the baseline and endline assessments.

**Table 1:** Respondents in baseline and endline surveys and in-depth interviews

	Kenya	Malawi
Baseline assessment: survey	47	46
Baseline assessment: in-depth interviews	15	13
Endline assessment: survey	39	53
Endline assessment: in-depth interviews	12	20
Total	113	132

The semi-structured questionnaire was mainly used for the technical staff (mid-level policy-makers), whereas the in-depth interviews, which also used a semi-structured guide, were conducted for the top-level policy-makers and senior researchers. Whereas for the mid-level policy-makers, the focus of the interview was on assessing individual EIDM skills and practice (including sourcing and accessing research evidence, appraising evidence quality, synthesising and applying evidence in decision-making) as well as institutional support mechanisms that enable or hinder EIDM practice, for the top-level policy-makers the focus was on institutional capacities (status, opportunities and challenges) and their perception of the EIDM technical capacities among MoH staff. Quantitative data from the baseline and endline assessments were analysed using descriptive statistics, whereas qualitative data were analysed using a thematic framework where data were classified into themes and sub-themes.

The second was implementation of activity-specific tools to assess activity effectiveness in contributing to the objectives of the intervention. These included the pre–post tests for the training workshops, feedback forms for the science-policy cafés, and documentation of effects of the various advocacy meetings conducted. The data that this paper has been drawn from reports of the project baseline and endline assessments, pre–post tests of training workshops, activity feedback forms and project progress reports that documented project outputs and changes in awareness, knowledge, attitudes and practices relating to EIDM.

#### External monitoring, learning and evaluation of the intervention

DFID commissioned external evaluation of the SECURE Health project that was implemented alongside project implementation (i.e. prospective evaluation). The evaluation used a realist approach and was conducted in three stages (in 2015, 2016 and 2017) (see [18, 21, 22]). The results of this evaluation have also provided data for this paper.

### Ethics review and approval

Ethics review and approval for the project’s research component was provided by the Kenya Medical Research Institute (KEMRI)’s Scientific and Ethics Review Committee whereas, in Malawi, this was provided by the College of Medicine Research and Ethics Committee.

### Intervention design

The SECURE Health project aimed to optimise individual and institutional capacity to use research in health sector decision-making in Kenya and Malawi. The design of the project’s intervention, back in 2013, was informed by a synthesis framework that combined elements of the UNDP’s 2009 framework on capacity development [23] and the WHO’s 2007 report on developing capacity for research use [8] (Table 2). The UNDP framework argued that capacity development efforts should focus on three areas, namely individual, institutional and organisational, to be effective. Additionally, the WHO report proposed the need for capacity development efforts aimed at enabling increased use of health to focus on increasing the supply of health research, increasing policy-makers’ capacity to source and use health research, and improving networking among researchers and policy-makers [8].

**Table 2:** Synthesis capacity development framework that informed intervention design

	Increase individual capacity	Improve institutional capacity	Enhance organisational capacity
Increase supply of policy-relevant research	Support development of policy briefs, systematic reviews	Provide policy briefs, systematic reviews in an ‘easy-to-access’ online databases Promote joint research priority-setting exercises	Build health research capacity in, or close to policy organisations (establish health systems research units in health ministries or in organisations with links to ministries)
Enhance capacity of policy-makers to source and use research	Provide training or mentoring in use of research evidence, commissioning of research studies and briefs Create stronger incentives for evidence use (e.g. through performance assessments, staff appraisals and leadership programmes)	Secure funding or raise government revenues to support development of policy analysis units, or perhaps research units within government bodies Improve access to research resources through improved Internet access, development of low-cost databases of research evidence (such as Hinari) Enhance leadership for demand and use of evidence	Develop and support knowledge broker capacity (establish knowledge broker organisations and networks)
Increase networking among policy-makers and researchers	Conduct special events or meetings that bring key actors together Require policy-maker participation in research (i.e. co-production of research)^a^	Establish institutional mechanisms that promote exchange between research and policy worlds Establish norms and regulations for research use (e.g. support legislation that requires publication of evidence base for new policies, integrate operational research and evaluation into existing processes and programmes)	Encourage mechanisms that bring technical expertise into government

Source: UNDP 2009 [23]; WHO 2007 [8]

^a^Authors’ additions

The intervention (Table 3) was limited by the funder’s requirements and did not therefore include activities in all the components of the framework above. For instance, the funding call focused only on the ‘demand-side’ of the research-to-policy continuum, and therefore the intervention did not include ‘supply-side’ activities in order to fit in the funder’s requirements. Further, the intervention did not focus on the broader organisational capacities due to resource limitations. The rationale for the intervention focus was the recognition of the need for joined-up system efforts that simultaneously build individual as well as institutional capacity for evidence use since the two (individual and institutional capacities) work in synergy. The intervention, therefore, had two objectives – to optimise institutional leadership and capacity to enhance evidence use in policy-making in the health sector and to enhance individual skills and capacity of policy-makers in the MoH and the legislature in accessing, appraising, synthesising and using evidence. The expected overall outcome of the intervention was an increased demand and use of research evidence in health sector decision-making in Kenya and Malawi.

**Table 3:** Intervention implemented

	Increase individual capacity	Improve institutional capacity	Enhance organisational capacity
Increase supply of policy-relevant research	*None as the focus of the funding call was on the ‘demand-side’ of the research-to-policy continuum and not the ‘supply-side’*		
Enhance capacity of policy-makers to source and use research	1. Train MoH technical staff in sourcing, appraising, synthesising and applying evidence in decision-making (5-day residential workshop) 2. Mentor and support-trained MoH staff in applying knowledge and skills acquired from the workshop above over 12-months (one-on-one monthly follow-ups, and 1-day refresher workshops every quarter)	1. Sustained advocacy with MoH top leaders on EIDM (one-on-one meetings with leaders, and annual national and regional forums with leaders on research-to-policy) to increase awareness on the important role of evidence in decision-making, and need to address institutional barriers to evidence use 2. Develop guidelines for evidence use 3. Develop health research agenda in Kenya 4. Review health research agenda in Malawi 5. Support MoHs to revive their defunct libraries, improve Internet connectivity, and subscribe to freely available databases such as Hinari	*None as our intervention was resource-limited and could therefore not focus on developing broader organisational capacities*
Increase networking among policy-makers and researchers		6. Support MoH research departments to introduce regular science-policy cafés to discuss research on urgent health issues; cafés were conducted every 6 months; 8 cafés in Kenya, 5 in Malawi	

*EIDM* evidence-informed decision-making, *MoH* Ministry of Health. Text in italics indicates the components of the concpetual framework where the project did not implement any interventions

While the intervention was designed through consultations with MoH leadership in both countries before the capacity needs assessments above, the results of these needs assessments informed the refinement of the intervention and detailed design of specific intervention activities. The main barriers to evidence use revealed by the needs assessments in both countries included little interest in using research evidence among top-level decision-makers due to competing political and personal interests; inadequate technical skills among MoH staff to access, appraise, analyse, synthesise and apply research evidence; inadequate time to access and use research evidence due to competing demands; lack of a mechanism for accessing research evidence (no repository); weak institutional linkages with research institutions; poor data quality and an inefficient health information system; and inadequate funding to support the generation and use of research evidence in decision-making. Inadequate staffing as a barrier to evidence use was only mentioned in the Malawi MoH but not in the Kenya MoH. Given that results of the needs assessment did not differ greatly between the two countries, similar intervention activities were implemented in Kenya and Malawi. Table 3 below presents the intervention implemented as it maps on the synthesis framework above.

Recognising the complexity of policy-making in government institutions, and the need for capacity-building efforts to take these into consideration, the intervention was implemented in partnership with the MoHs in the two countries. This provided ownership of the intervention by the MoHs. The institutions involved in the design and implementation of the intervention with the MoHs included the African Institute for Development Policy, Family Health International 360, the Consortium for National Health Research (Kenya), Malawi College of Medicine, and the East, Central and Southern Africa Health Community. In Kenya, the intervention was embedded in the work-plan of the then newly formed Research and Development Division in the MoH whereas, in Malawi, the intervention was embedded in the work-plan of the Malawi Knowledge Translation Platform (KTP) coordinated by the MoH’s Research Department.

## Results

### EIDM landscape in the health sector in Kenya and Malawi

The section summarises the EIDM landscape in the health sector in Kenya and Malawi as revealed by the capacity needs assessment conducted at the start of the intervention. At the national level in both Kenya and Malawi, there is no deliberate policy requiring evidence use or focused on promoting and enabling evidence use in decision-making in public policy and programming. There is some notable focus on the generation of research and data in both countries, but not as much focus on the translation of the research and data, and its actual use in decision-making. In regard to research, the Kenya MoH has KEMRI, mandated to provide the research the MoH needs for decision-making. While the Malawi MoH has no dedicated research institute, it expects universities (specifically the colleges of medicine) to provide the research needed for decision-making. Both MoHs have the District Health Information System II, which provides data from a country’s healthcare system supposed to inform decisions. However, funding for research generation and District Health Information System II is largely left to development partners, with the governments only investing in the salaries of researchers at KEMRI and universities. Through the National Commission for Science, Technology and Innovation in Kenya and the National Council for Science and Technology in Malawi, the two governments provide some small grants for research, including health research.

In 2012, the Malawi MoH established a KTP mandated to build capacity in evidence synthesis for health sector actors [24]. However, the work of the KTP has been hampered by limited funding. In 2013, the Kenyan government restructured the MoH and introduced, for the first time, a Division of Research and Development in July 2013 to promote and support MoH’s use of research evidence. There exist weak institutional linkages between the MoHs and the research institutions. For instance, in 2013, the Kenya MoH and KEMRI had no formal mechanisms required to enable sustained interaction and discussion of MoH’s research needs and/or KEMRI’s emerging research.

### Effect of intervention on institutional capacity

#### Effects on institutional tools, structures and mechanisms for enabling EIDM

The programme supported the two MoHs to develop “*guidelines for evidence-informed policy-making*” as an institutional tool for promoting and enabling evidence use. Although the plan was to develop and launch guidelines in the first 2 years and use the final year of project implementation to assess their usefulness, this was not possible due to delays occasioned by the many stages of review and approval for the guidelines. The guidelines were only adopted and launched in the last 4 months of project implementation, leaving no time to track how they were used. The key indicators that we agreed on with the MoH leadership to use in assessing commitment to institutionalisation and use of the guidelines include extensive dissemination, incorporating the guidelines into the orientation programme for new staff and requiring the use of guidelines in the processes and procedures of developing and providing policy advice. In Kenya, the development of the EIDM guidelines stimulated the MoH to appreciate the need for guidelines for the policy development process, which the ministry developed.

In addition to the guidelines, the MoHs adopted the EIDM training curriculum developed and used in the intervention as their institutional tool for developing EIDM capacity. The adopted curriculum incorporated lessons learnt in using the EIDM curriculum in the Kenya and Malawi trainings. Since 2016, the Kenya MoH has worked with several partners (including Amref Health Africa and KEMRI) to use the curriculum to train its staff working at country levels.

In order to define government’s research needs to guide the generation of responsive research, the intervention worked with the Kenya MoH to develop health research policy and priorities. The MoH led the development of the Research-for-Health Policy Framework and Priorities, with technical and financial inputs from our intervention. The process experienced notable delays that by the end of our intervention, the Research-for-Health Policy Framework and Priorities were fully developed, but yet to be adopted by the MoH pending health stakeholder review and approval. The draft policy outlines a comprehensive framework for, among others, improving and enabling the translation of health research for decision-making. It provides for the development of a knowledge management and translation framework that includes provisions for regular setting of priorities for research for health; the establishment of a functional knowledge-sharing mechanism comprising an online knowledge-sharing platform and regular conferences and dialogues for exchange of information and new research results; and the development of mechanisms for knowledge synthesis and exchange to inform policy and practice. The impact of this policy will be seen once the MoH adopts and implements it.

In Malawi, the intervention undertook a mid-term review of the implementation of the National Health Research Agenda adopted by the MoH in 2011 to generate lessons for improving its implementation. The review revealed that, except for one objective, the Agenda was failing to achieve most of its objectives. The only objective where progress was being made was on enabling the generation of research that is responsive to the country’s health priorities identified by the Agenda. The poor performance of the Agenda was mainly attributed to the lack of a comprehensive implementation plan and committed resources for implementing it. The MoH discussed the results and recommendations of the review with stakeholders in order to generate support from other government agencies and development partners in implementing the Agenda. Although the endline survey did not show much change in the implementation of the Agenda, the impact of this review was not expected to be immediate and will likely be seen in years to come.

The regular science-policy cafés were meant to provide a framework for strengthening interactions for policy-makers and researchers. Because the cafés were largely attended by different groups of researchers given the issue of focus, our endline survey did not find evidence that they improved relationships between researchers and policy-makers. However, endline results showed that the cafés were seen as one of the most effective activities implemented by the intervention because they facilitated critical discussions of evidence on urgent policy issues. Further, some of the evidence and recommendations provided by the cafés informed policy decisions in both Kenya and Malawi MoHs. In Kenya, the café on providing free-of-charge maternal health services informed the country’s policy shift on its funding mechanism for the free-of-charge maternity health services policy. In Malawi, the café on healthcare financing informed the MoH’s decision to retain its policy on user fee waivers for the provision of health services. The café on youth sexual and reproductive health and the role of sexuality education stimulated critical conversations between the MoH and Ministry of Education on providing comprehensive sexuality education in Malawi. Similarly, the recommendations from the café on bridging the health research-to-policy gap in Malawi stimulated efforts within the MoH and parliament to push for increased government funding for research, including health research. However, our hope to see the two MoHs institutionalise the cafés as formal platforms for sustained interactions between policy-makers and researchers was not realised by the end of the project.

Regarding strengthening linkages at institutional level, the intervention stimulated closer working relations between the MoH and KEMRI in Kenya, and the MoH and the College of Medicine in Malawi. The interventions further provided ideas on how these institutions could work more collaboratively to promote the use of existing research evidence in policy and programming decisions. Although the benefits of these strengthened relationships were not immediately demonstrable in improving the use of research evidence, these may be seen in the future.

In relation to improving structures for enabling EIDM, the project sought to stimulate the two MoHs to revive their defunct libraries, improve Internet connectivity and get subscribed to journal databases that are freely available such as the WHO’s Hinari Access to Research for Health Programme. Unfortunately, these efforts bore limited results. The process of getting connected to the WHO Hinari database was initiated by the Kenya MoH but was not completed by the end of the intervention. For the Malawi MoH, subscription to Hinari was not initiated as the Internet connectivity remained severely poor throughout intervention implementation.

#### Effects on resources: funding and personnel for enabling evidence use

Having adequate financial and human resources is critical for enabling evidence use. Following the project’s high-level advocacy, the Kenya MoH introduced a budget line for the Research and Development unit in the 2015/2016 financial year to enable the unit to implement activities that facilitate evidence use in the ministry. For Malawi, the project did not stimulate any increases in funding for enabling evidence-informed policy-making mainly because, throughout the project period, the government of Malawi was operating entirely on an ‘austerity budget’, and so it was clear from the start of the project that it was going to be difficult for the government to increase or introduce budget lines aimed at enabling evidence-informed policy-making. Nevertheless, the intervention stimulated new thinking from other actors in the country, which has resulted in more funding from development partners through the MoH for EIDM [25].

Regarding personnel, results of the baseline identified inadequate numbers of staff, and weak or lacking skills in EIDM as among the key barriers to evidence use. Therefore, while the training programme (discussed in the next section) aimed at building skills in EIDM for existing staff, our advocacy efforts hoped to stimulate recruitment of more staff to support EIDM work. In both countries, the ministries reported to have frozen new recruitments throughout the project period, and so the advocacy did not result in new recruitments. However, the Research unit in the Malawi MoH received two additional staff through transfers from other departments during the implementation of the project, increasing its staffing from two to four. In Kenya, the opposite occurred, where four of the existing staff in the Research unit left to join other divisions and one retired, leaving only one staff member by the end of the project.

### Effect of intervention on individual capacity

#### Effect of policy-maker training and mentorship on EIDM capacity

Results of the external evaluation of the SECURE Health training programme revealed “*strong evidence that the training and mentorship activities had increased participants’ awareness, technical knowledge and or skills*” ([18], p. 36). These results confirmed our own internal evaluation, which also showed an increase in technical capacity to use research in policy-making and programme implementation. Table 4 summarises self-ratings by MoH technical staff who benefited from the EIDM training in key EIDM competencies using a Likert scale, with 1 being the lowest and 5 the highest level of knowledge and skill. This self-rating was done at three points, the first one immediately before the training workshop, the second one at the end of the workshop, and the third one at the end of the intervention, which was about 18 months after the 5-day training workshop.

**Table 4:** Average ratings of evidence-informed decision-making skills at pre–post training, immediately after training and at endline

Skills/knowledge average rating	Kenya				Malawi			
	Pre-test	Post-test 1	Endline	Point change^a^	Pre-test	Post-test 1	End line	Point change^a^
Accessing research, including developing a search strategy and searching for specific information	2.78	4.32	4.04	1.26	3.14	4.43	4.54	1.40
Defining and developing a policy question	2.74	4.36	4.08	1.34	2.50	4.57	4.50	2.00
Using free databases	2.11	3.93	4.04	1.93	2.50	4.33	4.46	1.96
Using advanced Google searching	2.85	4.11	4.17	1.32	3.27	4.48	4.38	1.11
Appraising evidence	2.7	4.04	3.75	1.05	2.71	4.43	4.50	1.79
Synthesising evidence	2.85	3.82	3.79	0.94	2.64	4.29	4.31	1.67
Applying evidence	2.93	4.04	4.21	1.28	2.50	4.48	4.71	2.21
Linking with research institutions and researchers	2.26	3.85	3.13	0.87	2.14	4.05	4.14	2.00
Average score	2.65	4.06	3.90	1.25	2.68	4.38	4.44	1.77

^a^Point change is calculated for endline scores onlyF[

As seen in the table, the average ratings for research use skills among the Kenya MoH training participants improved from 2.65 before the training to 4.06 immediately after the training and 3.90 at endline. Similarly, among the Malawi MoH training participants, there was an improvement in the average ratings for research use skills from 2.68 before the training to 4.38 immediately after the training and 4.44 at endline. Besides assessing the change in perceived research use skills among trained staff, we also used the pre–post test survey to assess the EIDM knowledge acquired through the training. Results showed notable improvements in knowledge on various EIDM components, including the complexity of the policy-making process, the role and place of different types of research evidence in the policy-making process, barriers to evidence use and ways of overcoming these.

An important aspect of the training programme’s follow-up support and mentorship was to support actual on-the-job application of skills, and the completion of policy briefs that learners started preparing during the 5-day initial training workshop over 12 months. On-the-job support proved difficult as trainees were very busy and either had very short turn-around times for activities that required the inputs of the trainers and so trainers were not able to support them, or learners were not forthcoming in seeking the support of trainers in real job tasks. As a result, most of the follow-up focused on supporting learners to complete their policy briefs on issues they had identified as urgent for their units/divisions prior to the training. For Kenya, of the 34 staff, 12 completed and disseminated individual or group policy briefs, whereas for Malawi, out of 26 learners, 15 completed and disseminated individual policy briefs. For those who were unable to complete their policy briefs, the main reason cited was lack of time due to busy schedules.

The development of policy briefs by trained staff provided a more objective way of gauging skills acquired by learners in the EIDM process. From our experience in both countries, except for a few briefs that were of good quality, most of the briefs developed were of average quality, pointing to the need for the technical officers to do more practice in order to improve the quality of briefs they develop. One of the MoH staff in Malawi shared her policy brief with a funding agency, which in turn provided funds to tackle the issue highlighted in the brief. Her brief advocated for the decentralisation of HIV testing to reduce results turnaround time through the use of point of care HIV diagnostic devises. Another learner in Kenya got his policy brief to inform the MoH policy/strategy on water and sanitation; his brief advocated for timely water safety surveillance highlighting the burden and cost of failing to do so.

#### Effect of skills development on EIDM practice

The 2016 external evaluation report ([22], p. 12) and our own internal evaluations showed that the training and mentorship components increased the confidence and improved the attitudes of MoH staff towards EIDM. Some of the trained staff became evidence champions in their divisions and units. For example, a senior officer in the Kenya MoH’s Standards and Quality Assurance division who benefited from the training trained the technical staff working under him on evidence use. Interviews with trained staff and MoH leadership revealed specific examples of how the training and mentorship, and other project activities had impacted their practice of EIDM in the MoH in the two countries, as captured below:

“*That training has helped me focus on evidence in my work. I attended the training when we were in the process of formulating the school health and nutrition policy. So that knowledge helped me very much in accessing the evidence on school health and nutrition that was reviewed for the policy*.” Malawi MoH Staff, 2016.

“*The training enhanced my confidence that I can lead in the process of coming up with an evidence-informed policy. From the policy brief I prepared, we have put together a few technical people to finalise it and turn it into a concept note that we will later on develop into a policy*.” Malawi MoH Staff, 2016.

“*The project has stimulated a new way of thinking in the staff involved. Now we need to make what the project has initiated a norm in the Ministry. We need to ensure that EIDP* [evidence-informed decision-making and policy] *is the norm*.” Kenya Senior MoH Official, 2017.

The external evaluation found evidence that “*a number of trainees were using their skills to inform policies or generate evidence products, and that BCURE had made an important contribution to this, alongside including donor-supported programmes providing an opportunity for evidence use*” ([18], p. 45). For the staff who reported increased evidence use following the training, their motivation was reported as strong personal interests, even a passion, in working with evidence and opportunities in their roles to use evidence [22].

The evidence use by many of the trained staff, however, did not translate into evidence use as routine practice within the MoHs. Our endline survey, conducted with a sample of trained staff as well as those who did not go through the training showed no change in EIDM practice within the MoHs in the two countries. Specifically, the endline survey results revealed no notable improvement in the average frequency of evidence use at endline relative to the baseline results. Results of the external evaluation confirmed these results, noting that “*There is strong evidence that BCURE participants in Kenya … all acquired new knowledge and skills that led to some degree of behaviour change, although as yet there is limited indication of routine evidence use among trainees*” ([18], p. 46).

In our internal evaluation, MoH staff in both countries identified their most severe barrier to evidence use at both baseline and endline as the lack of relevant research evidence and/or the lack of access to evidence. This is not surprising since the project’s efforts at institutional level to stimulate leaders into action to address persisting barriers to accessing research evidence were not very effective as already discussed. The fact that the intervention tried to address the ‘access to evidence’ barrier by getting the Kenya MoH connected to Hinari but failed shows the disconnect within the MoH in steering the intervention; the staff responsible for connecting MoH to Hinari were different from the staff facing the challenge of accessing evidence, and therefore the lack of urgency in completing this task reflected the low appreciation of common goals among staff at the MoH. Additionally, while the intervention strengthened links between the MoH and existing research institutions (KEMRI in Kenya and College of Medicine in Malawi) through its activities, the benefits of these strengthened relationships were not immediate in improving access to research evidence, but may be seen in the future.

### Challenges faced during implementation

#### Complexity in institutional arrangements and power relations within bureaucracies

We experienced challenges arising from the complex institutional structures and arrangements compounded by a web of power relations among individuals within these structures. In Kenya, for instance, the Research and Development unit was a Division at the start of the project in 2013, and this gave it a higher level in the hierarchy. Within 2 years, this had been demoted to a Unit, a decision occasioned largely by the exit of a top leader who valued research more than his successor.

#### Slow processes, weak accountabilities and MoH staff inertia

Co-implementing project with MoH staff exposed us to the slow processes, weak or lacking accountability structures, and inertia among MoH staff that often resulted in delays in implementing project activities. For instance, the process of getting the Kenya MoH subscribed to Hinari took a much longer period partly because the MoH staff responsible for this often did not do what was agreed upon, requiring a lot of follow-ups. Therefore, a process that appeared quite straight-forward, was not completed by the end of the project.

#### Frequent staff transfers and turnovers

Throughout the 3 years of implementing the project, there were numerous staff changes in the two MoHs. Such frequent staff transfers undermined project implementation, often leading to delays in activity implementation or low priority accorded to the project altogether by incoming staff.

#### Challenges with selecting the staff to benefit from the training and mentorship programme

In order to attract the right and motivated MoH staff to benefit from the training, we advertised the training within MoH and required interested staff to apply for the training. Although we aimed to select 30 MoH staff for the training in each country, we received few applications to select from (26 applications in Kenya and 15 in Malawi). To address this challenge, we engaged the MoH leadership to review the list of applications as well as devise other ways of identifying the right staff for the training. The MoH leadership in both countries asked heads of divisions to nominate relevant staff from their divisions for the training, which led to more applications. In Kenya, the MoH further revised the list of those who had applied, dropping some of them, who they argued were always attending trainings but failing to deliver in their work.

#### The foundational training workshop was ‘too intense’

The main complaint from MoH staff about the foundational 5-day EIDM training workshop was that it was ‘too intense’ and the time was not adequate. Although we considered offering the training in a series of modules over a month, we realised that we would face the challenge of getting different staff from the MoH depending on availability for different modules. As such, the option of a 5-day intense foundational workshop was made to ensure that all selected staff go through all the modules. It was also not possible to spread the training over periods longer than 1 week as staff would not be allowed to be away for such long periods.

#### Poor participation in the training’s follow-up mentorship programme

There was low participation in the one-on-one follow-up sessions and refresher workshops as well as slow progress towards completion of policy briefs. We engaged MoH leadership to support the process and require the completion of policy briefs. This strategy helped to get more learners actively involved in the mentorship process. We changed strategy to have learners work in groups to produce policy briefs, but this worked for some groups and not for others, and forced some learners to revert to working on individual briefs. We also increased the frequency of one-on-one contact with every learner from every 2 months to every 1 month to better understand the challenges they faced and provide timely feedback on draft policy briefs. This meant that we invested more personnel time than was budgeted for.

## Discussion

There is increasing interest and investment in building capacity on the demand-side of evidence for enabling increased use of evidence in decision-making (see [9–12, 20, 26]). Yet, evidence on the effectiveness of capacity-building interventions for evidence use in resource-poor settings remains sparse [12]. This paper’s discussion of the results of one of the DFID-funded BCURE projects in Kenya and Malawi presents valuable knowledge for informing the growing efforts in this area and the emerging thinking around effective capacity development for increased use of evidence in decision-making. While the synthesis framework that informed the intervention design provides broader guidance on important intervention areas to ensure effectiveness in EIDM capacity development, results shared in this paper not only deepen the understanding of intervention design and why it may work or fail, but also point to ways of enriching the framework.

At an individual level, the training and mentorship for MoH staff increased the knowledge and skills in the use of evidence, which confirms results from other studies (see [12, 27]) on the effectiveness of this activity. There are various insights we can draw on from this experience in building individual capacity for evidence use. First, is the importance of involving top-level policy-makers in the selection of technical staff to be trained, which ensures that the right staff are selected, but also generates demand for the use of the skills acquired from the training; the need to select strategic individuals who can cascade the skills learned to other staff in their divisions and units is important. Additionally, the need to identify individuals with a genuine motivation for evidence use is critical as these are the individuals who, in this case, became evidence champions in their divisions and units, and therefore sustained and championed the EIDM practice. The importance of training a ‘critical mass’ of individuals in a department or division has been shown as important in cascading and sustaining the EIDM practice [12, 18]. While in our intervention we trained and mentored individuals dispersed in different departments and divisions in the MoH in both countries, future interventions may achieve more if they target a group of technical staff working in one department or division or on a particular programme. Finally, keeping trained staff connected to share experiences and lessons was shown to be important in sustaining practice [18].

The question of sustainability of such a training programme is an important one, and which, because of the short-term nature of our intervention, was not addressed. While we made the EIDM training curriculum used by the programme available as open access for use by other actors, getting the EIDM training programme incorporated into existing pre-service and in-service training programmes for civil servants provides a more effective way to enable sustained capacity development for EIDM. Beyond the project period, the authors have continued to engage relevant training institutions in Kenya and Malawi to review and incorporate the EIDM training curriculum in their existing programmes.

In relation to strengthening institutional capacity for evidence use, the intervention’s average results confirm the well-recognised fact that strengthening institutional capacity is most critical in capacity development efforts, but it is complex, has many dimensions, and requires sustained political commitment and investments over a long period of time [7, 12, 14]. For instance, in regard to funding for EIDM, the Kenya MoH introduced a budget line for funding the activities of its Research and Development Division. During the same period, this ‘division’ was demoted to a ‘unit’, thus lowering its stature within the MoH hierarchy. It also experienced severe staffing shortages when its key staff were either transferred to other departments or retired without replacement. This represented a mismatch between what the intervention was seeking to achieve (i.e. strengthening institutional capacity for evidence use) and what the new leadership in the MoH was doing. Yanguas [28] has argued that public sector reform programmes take root in places where there is a match between the ideas being proposed and the norms and incentives of the ruling elite. Our intervention shows that, in contexts where elites change frequently (like the Kenya MoH), there is a need for sustained political economy analysis and engagement with the incoming elites as well as of rethinking interventions to mitigate the negative impact occasioned by frequent changes of elites.

The guidelines for evidence use developed by the project and adopted by both MoHs provide an important tool for promoting and embedding EIDM in MoH processes if staff are trained on how to use them in their work [12]. Hawkes et al. [12] have argued that guidelines need to be underpinned by regulatory frameworks that enforce their use. They gave the example of the guidelines developed by the United Kingdom’s National Institute for Health and Care Excellence (NICE), arguing that these guidelines are actually used in the United Kingdom health system because of the existing regulatory framework, which requires the health system to use these guidelines. In our case, the project ended before the guidelines could be incorporated into institutional processes that would require their use, pointing to the importance of longer-term initiatives when it comes to strengthening institutions [14].

The results of the science-policy cafés challenge the argument by other scholars (see [29–31]) that these improve relations between policy-makers and researchers. The difference may be in the design of the dialogues; in our case, each dialogue focused on a different issue, which means it convened different policy-makers and researchers, providing limited opportunity to build relations. If designed this way, our results suggest that dialogues will be most useful in enabling discussion of evidence and informing specific policy decisions but may not necessarily nurture relations. Furthermore, the dialogues informed policy decisions on issues that were already high on the political agenda, demonstrating the importance of linking or focusing EIDM efforts on policy issues that are high on the political agenda [32]. The failure to get the MoHs to institutionalise science-policy cafés points to the need for future efforts to get formal high-level commitment to sustaining government investment in institutional strengthening processes beyond donor-funded periods at the start of projects.

Embedding the intervention in the Research division/department within the two MoHs was a critical success factor, confirming the findings of other scholars [33, 34]. Even with this, the intervention was still negatively affected by changing interests and power relations, and weak accountabilities and slow processes within the MoHs. This points to the importance of sustained political economy analysis before and during implementation, as recommended by other scholars [7, 12, 18]. This would, among others, ensure implementers of interventions are constantly in touch with top-level leaders at the MoH to generate their support for interventions and to ensure interventions align with shifting priorities. Given the importance of generating and sustaining top-level ownership and commitment, and aligning interventions to changing interests and shifting power relations within bureaucracies, we suggest that conceptual frameworks that guide future interventions in EIDM capacity development for civil servants in low- and middle-income countries should reflect these factors.

The two MoHs’ over-reliance on funding from development partners for the health sector is an important factor that shapes EIDM practice. The average priority that the intervention received in both MoHs was partly because it was not a priority of many of the funders investing heavily in these two MoHs. This means that implementers of EIDM initiatives in future need to also sustain engagement with key funders for the MoHs so that these actors support the intervention in their conversations with the leadership of the MoHs and through additional funding.

Overall, the results of the intervention confirm the important need for governments, funders and other development actors to take holistic and longer-term approaches to capacity development efforts for increased research use. Simultaneous and sustained efforts that address both ‘supply-side’ and ‘demand-side’ capacity challenges at individual, institutional and organisational levels could be more effective. Hawkes et al. [12] have proposed a framework for measuring impact of capacity strengthening efforts in health policy-making. Our experience shows that the design of capacity strengthening interventions for evidence use in health policy-making needs to be holistic if such a framework is to be useful in assessing intervention effectiveness.

## Conclusions

Among the many barriers to evidence use, weak capacity for evidence use in policy and programme decisions has attracted much focus in the last decade [4, 8–12]. In order to inform and enrich the ongoing and future efforts to strengthen capacity for evidence use, this paper has discussed experiences and lessons from an intervention implemented in Kenya and Malawi to strengthen individual and institutional capacity for evidence use within the MoHs. The results discussed in the paper have shown that training and mentorship programmes in evidence use can be effective in improving the competencies of civil servants in EIDM. However, such programmes need to be incorporated in existing pre-service and in-service training programmes in order to train a critical mass of civil servants and to be sustainable. On strengthening institutional capacity for evidence use, the paper’s results show the difficulty and complexity of such efforts and the critical need for sustained political commitment and long-term investments. The paper’s results suggest that future efforts would be more effective if they focused on incorporating training and mentorships for EIDM in existing programmes for sustainability and if they took a long-term, politically driven focus on strengthening institutional capacity for EIDM.

## Data Availability

The datasets used and/or analysed during the current study are available from the corresponding author on reasonable request.

## Abbreviations

BCURE: Building Capacity to Use Research Evidence
DFID: United Kingdom’s Department for International Development
EIDM: Evidence-Informed Decision-Making
KEMRI: Kenya Medical Research Institute
KTP: Knowledge Translation Platform
MoH: Ministry of Health
SECURE Health: Strengthening Capacity to Use Research Evidence in Health Policy
UNDP: United Nations Development Programme

## References

[1] Stewart R, Langer L, Erasmus Y (2018). An integrated model for increasing the use of evidence by decision-makers for improved development. Dev South Afr.

[2] Rutter J, Gold J. Show Your Workings: Assessing How Government Uses Evidence to Make Policy. London: Institute for Government; 2015. https://www.instituteforgovernment.org.uk/sites/default/files/publications/4545%20IFG%20-%20Showing%20your%20workings%20v8b.pdf. Accessed 31 Dec 2018.

[3] Jones H, Jones N, Shaxson L, Walker D (2012). Knowledge, Policy and Power in International Development: A Practical Guide.

[4] Newman K, Fisher C, Shaxson L (2012). Stimulating demand for research evidence: what role for capacity building. IDS Bull.

[5] Chalmers I (2005). If evidence-informed policy works in practice, does it matter if it doesn’t work in theory?. Evid Policy.

[6] Buse K, Mays N, Walt G (2012). Making Health Policy.

[7] Cairney P (2016). The Politics of Evidence-Based Policy Making.

[8] World Health Organization, Alliance for Health Policy and Systems Research. Sound Choices: Enhancing Capacity for Evidence-Informed Health Policy. Geneva: WHO; 2007. https://www.who.int/alliance-hpsr/resources/publications/9789241595902/en/. Accessed 30 Oct 2018.

[9] World Health Organization, Alliance for Health Policy and Systems Research, Wellcome Trust. Call for Expression of Interest: Heightening Institutional Capacity for Government Use of Health Research. Geneva: WHO; 2018. https://www.who.int/alliance-hpsr/callsforproposals/HIGH-Res-call-1-2.pdf. Accessed 24 Jun 2018.

[10] DFID. Building Capacity to Use Research Evidence (BCURE). 2014. https://bcureglobal.files.wordpress.com/2013/12/bcure-flyer-november-2014.pdf. Accessed 3 Oct 2018.

[11] DFID (2018). Strengthening the Use of Evidence for Development Impact (SEDI) (call for proposals).

[12] Hawkes S, Aulakh B, Jadeja N, Jimenez M, Buse K, Anwar I (2016). Strengthening capacity to apply health research evidence in policy making: experience from four countries. Health Policy Plan.

[13] UNDP. Capacity Development: Measuring Capacity: UNDP; 2010. p. 2. https://www.undp.org/content/undp/en/home/librarypage/capacity-building/undp-paper-on-measuring-capacity.html. Accessed 16 Oct 2018.

[14] Potter C, Brough R (2004). Systemic capacity building: a hierarchy of needs. Health Policy Plan.

[15] Walters H (2007). Capacity Development, Institutional Change and Theory of Change: What do we mean and where are the linkages..

[16] Liverani M, Hawkins B, Parkhurst J (2013). Political and Institutional Influences on the Use of Evidence in Public Health Policy. A Systematic Review. PLoS One.

[17] Oliver L, Innvaer S, Lorenc T, Woodman J, Thomas J (2014). A systematic review of barriers to and facilitators of the use of evidence by policymakers. BMC Health Serv Res.

[18] Vogel I, Punton M. Final Evaluation of the Building Capacity to Use Research Evidence (BCURE) Programme: Comparative Report. London: ITAD; 2018. https://itad.com/reports/final-evaluation-building-capacity-use-research-evidence-bcure-programme/. Accessed 30 Nov 2018.

[19] Clar C, Campbell S, Lisa D, Wendy G. What Are the Effects of Interventions to Improve the Uptake of Evidence from Health Research into Policy in Low and Middle-Income Countries? London: DFID; 2011. https://assets.publishing.service.gov.uk/media/57a08ab3ed915d3cfd0008c2/SR_EvidenceIntoPolicy_Graham_May2011_MinorEditsJuly2011.pdf. Accessed 16 Dec 2019.

[20] Hewlett Foundation. Evidence-Informed Policymaking Call for Proposals for African Policy Research Institutions to Advance Government Use of Evidence: Hewlett Foundation; 2018. https://hewlett.org/eipafrica/. Accessed 4 May 2018.

[21] Vogel I (2015). BCURE Programme Evaluation Report: Stage 1 Evaluation of the SECURE Health PRogramme.

[22] Vogel I, Oduor A (2016). BCURE Programme Evaluation Report: Stage 2 Evaluation of the SECURE Health Project.

[23] UNDP (2009). Capacity development: A UNDP Primer.

[24] Berman J, Mitambo C, Matanje-Mwagomba B, Khan S, Kachimanga C, Mwape L (2015). Building a knowledge translation platform in Malawi to support evidence-informed health policy. Health Res Policy Syst.

[25] Malawi-Liverpool-Wellcome Trust Clinical Research Centre. Policy Unit (web page). MLW. https://www.mlw.mw/index.php/research/policy-unit.html. Accessed 12 Dec 2018.

[26] World Health Organization. Call for Proposals: Leadership Development for Enhanced Decision-Making: WHO; 2012. https://www.who.int/alliance-hpsr/callsforproposals/alliancehpsr_irpcallleadership.pdf. Accessed 29 Dec 2018.

[27] Uneke C, Ezeoha A, Uro-Chukwu H, Ezeonu C, Ogbu O, Onwe F (2015). Enhancing the capacity of policy-makers to develop evidence-informed policy brief on infectious diseases of poverty in Nigeria. Int J Health Policy Manag.

[28] Yanguas P. Understanding the Paths and Politics of Change. Report No.: 28: Public Sector Reform in Africa; 2017. http://www.effective-states.org/public-sector-reform-in-africa-understanding-the-paths-and-politics-of-change/. Accessed 2 Jan 2019.

[29] El-Jardali F, Lavis J, Moat K, Pantoja T, Ataya N (2014). Capturing lessons learned from evidence-to-policy initiatives through structured reflection. Health Res Policy Syst.

[30] Dobbins M, Hanna S, Ciliska D, Manske S, Cameron R, Mercer S (2009). A randomized controlled trial evaluating the impact of knowledge translation and exchange strategies. Implement Sci.

[31] Traynor R, DeCorby K, Dobbins M (2014). Knowledge brokering in public health: a tale of two studies. Public Health.

[32] Andrews M, Pritchett L, Woolcock M. Doing Problem Driven Work: Harvard University: Harvard Kennedy School; 2015. Report No.: 307. https://bsc.cid.harvard.edu/files/bsc/files/doing_problem_driven_work_wp_307.pdf. Accessed 3 Jan 2019.

[33] Booth D, Unsworth S. Politically Smart, Locally Led Development. London: Overseas Development Institute; 2014. https://www.odi.org/sites/odi.org.uk/files/odi-assets/publications-opinion-files/9158.pdf. Accessed 3 Jan 2019.

[34] Faustino J, Booth D. Development Entrepreneurship: How Donors and Leaders can foster Institutional Change. London: Overseas Development Institute; 2014. Report No.: Case Study 2. https://www.odi.org/sites/odi.org.uk/files/odi-assets/publications-opinion-files/9384.pdf. Accessed 4 Jan 2019.

